# Exploring and retrieving sequence and metadata for species across the tree of life with NCBI Datasets

**DOI:** 10.1038/s41597-024-03571-y

**Published:** 2024-07-05

**Authors:** Nuala A. O’Leary, Eric Cox, J. Bradley Holmes, W. Ray Anderson, Robert Falk, Vichet Hem, Mirian T. N. Tsuchiya, Gregory D. Schuler, Xuan Zhang, John Torcivia, Anne Ketter, Laurie Breen, Jonathan Cothran, Hena Bajwa, Jovany Tinne, Peter A. Meric, Wratko Hlavina, Valerie A. Schneider

**Affiliations:** National Center for Biotechnology Information, National Library of Medicine, National Institutes of Health, Building 38A, 8600 Rockville Pike, Bethesda, MD 20894 USA

**Keywords:** Biotechnology, Computational biology and bioinformatics, Molecular biology

## Abstract

To explore complex biological questions, it is often necessary to access various data types from public data repositories. As the volume and complexity of biological sequence data grow, public repositories face significant challenges in ensuring that the data is easily discoverable and usable by the biological research community. To address these challenges, the National Center for Biotechnology Information (NCBI) has created NCBI Datasets. This resource provides straightforward, comprehensive, and scalable access to biological sequences, annotations, and metadata for a wide range of taxa. Following the FAIR (Findable, Accessible, Interoperable, and Reusable) data management principles, NCBI Datasets offers user-friendly web interfaces, command-line tools, and documented APIs, empowering researchers to access NCBI data seamlessly. The data is delivered as packages of sequences and metadata, thus facilitating improved data retrieval, sharing, and usability in research. Moreover, this data delivery method fosters effective data attribution and promotes its further reuse. This paper outlines the current scope of data accessible through NCBI Datasets and explains various options for exploring and downloading the data.

## Introduction

The National Center for Biotechnology Information (NCBI), a division of the National Library of Medicine (NLM), is responsible for archiving, preserving, and providing access to a vast amount of biological sequence data contributed by researchers and curated by NCBI staff. This extensive collection of sequence data for organisms across the tree of life has the potential to reveal deeper insights into evolutionary history and its implications for medicine and biological research^1^. However, accessing this data can be challenging. The rapid increase in sequencing data, driven by decreasing sequencing costs, the introduction of new data types and formats, and the expansion of metadata, has exceeded the capabilities of NCBI’s web and programmatic interfaces to provide easy and intuitive access to data^2^. Search capabilities should be more user-friendly, results should be easier to browse, and data downloads should be more comprehensive, consistent, and scalable. Moreover, the full potential of genomic ‘big data’ is unlocked only when researchers, bioinformaticians, and data scientists, are provided with extensive metadata that follows well-documented organizational schemas, accompanied by clear and well-defined connections between sequence data and its corresponding metadata. Explicit linkage between sequence data and its metadata facilitates improved reusability and proper attribution.

**Fig. 1:**
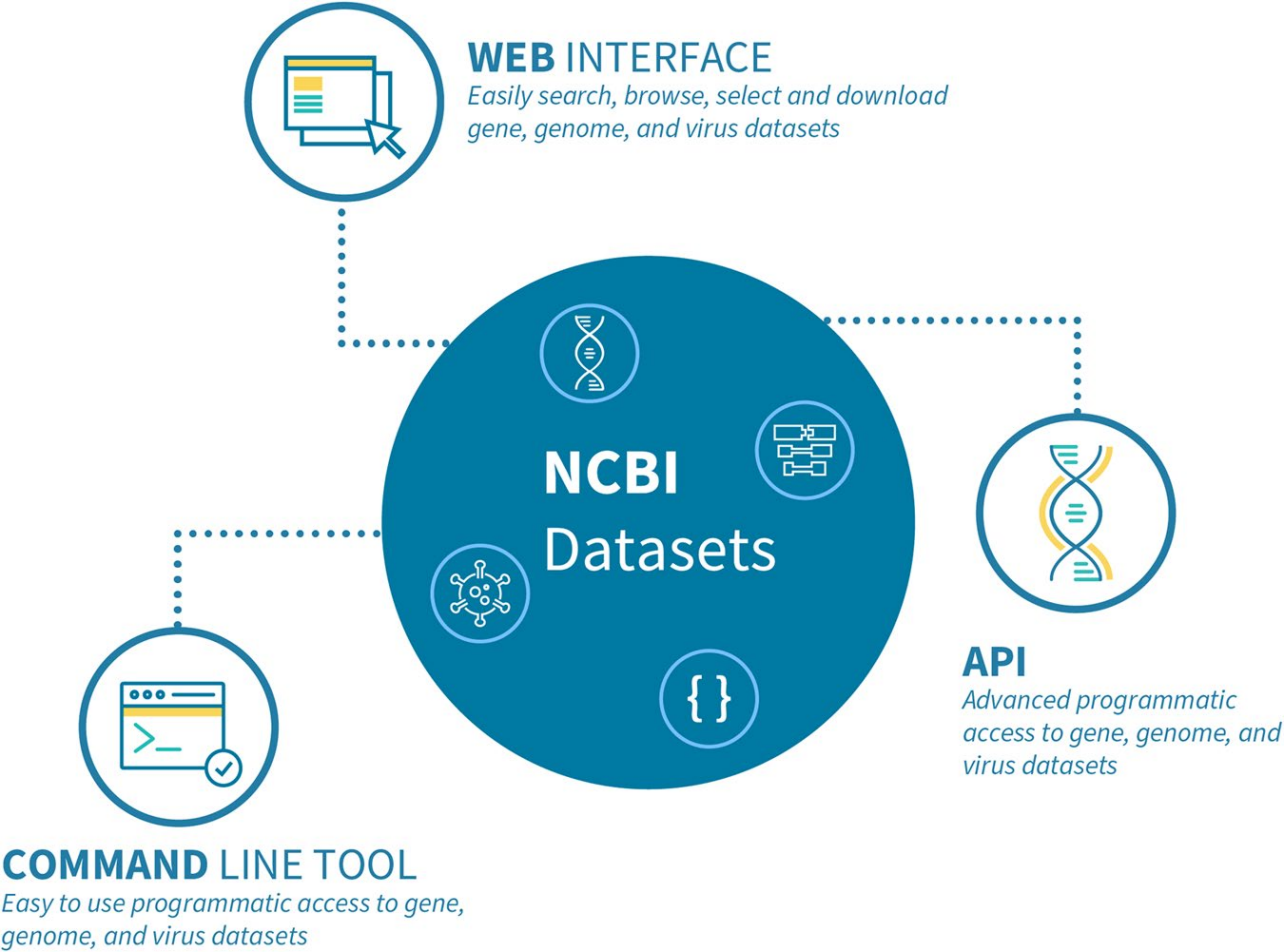
NCBI Datasets interfaces for data access: NCBI Datasets offers web, command-line, and API access points that facilitate the search for and download of genomic sequences, annotations, and comprehensive metadata. These tools, including web-based and programmatic interfaces, guarantee consistent data retrieval from NCBI Datasets.

In response to these challenges, NCBI launched NCBI Datasets (https://www.ncbi.nlm.nih.gov/datasets/), a resource that simplifies the discovery, exploration, and downloading of open-access biological sequences and metadata housed at NCBI. NCBI Datasets provides new interfaces for data access, including web interfaces, command-line tools and OpenAPI (https://www.ncbi.nlm.nih.gov/datasets/docs/v2/reference-docs/rest-api/) (Fig. 1), as well as a new data delivery system described in the Results section under “Data packages: a new data delivery system.” Recognizing the sheer volume of data we manage, we aim to facilitate scalable programmatic access while ensuring the tools remain accessible, even for those with limited technical experience. Our commitment is to develop solutions that not only efficiently process large datasets but are also easy to use, supporting a wide range of users in leveraging our data’s full potential regardless of their programming skills^3^.

A few fundamental principles guide us in achieving our goals and underscore our distinctive approach to data access and delivery.

- Adherence to FAIR principles: We ensure that data is easy to locate, use, and share, and readily available for scientific discovery^4^.
- Smart searches: We use intelligent search features that easily recognize commonly used terms like names of genes or species, access numbers, and other unique labels.
- Easy-to-use tools: We provide web-based and programmatic interfaces to facilitate access to the extensive biological information available at NCBI for all users, regardless of their expertise level.
- Delivery of data packages: We bundle sequence, annotation, and metadata, enhancing citation and attribution while reducing the need to retrieve data from multiple databases.
- Detailed documentation: We provide thorough documentation of our metadata schemas and command-line syntax, and offer extensive tutorials.

As we develop, NCBI Datasets is transitioning from the legacy Entrez web interfaces, including Assembly and Genome, to the new NCBI Datasets pages. This transition is being implemented gradually to allow users of the older web interfaces to acclimate and gain proficiency with the new system. We aim to ensure the change is seamless and user-friendly, enabling the biological research community to adjust its workflows to the new system. NCBI Datasets is designed to be flexible and readily incorporate new data types and metadata, positioning NCBI to meet the community’s requirements and remain sustainable. Engaging with the scientific community has been crucial in shaping our resources. NCBI Datasets continues to work closely with the biological research community through user interviews, integrating feedback, and utilizing analytics to refine our interfaces and improve the data standard we deliver. We adhere to an iterative and user-centered development approach to maintain our fundamental principles, ensuring our resources align with the needs and expectations of the community. This article outlines the diverse data types available via NCBI Datasets; illustrates the usage of our web and programmatic interfaces for querying and obtaining sequence data, annotations, and metadata; offers guidance on incorporating these datasets into active workflows; and points to comprehensive documentation for optimal practices. We conclude by sharing our visions for the future of NCBI Datasets and by highlighting upcoming data and services.

## Results

### Data packages: a new data delivery system

Accessing data using the existing NCBI interfaces requires understanding the structure of NCBI’s databases^5^. This involves understanding the kind of data each database holds, database update schedules, and how they link to data in other databases. Data downloading typically combines navigating various database record pages and associated FTP sites to acquire the desired sequence and its corresponding metadata. For example, to get the complete set of sequence data and metadata for an annotated genome assembly, a user would need to download genome sequence and annotation files from either a Genome or Assembly record, then obtain partial metadata from the Assembly and BioSample databases, and finally get information about annotation and genome quality analysis from a separate RefSeq annotation report page. The high cost of gathering and downloading data, including time, effort, and knowledge of NCBI databases, is a significant obstacle for new users and a source of frustration for even the most experienced users. These challenges motivated us to develop a more straightforward data delivery method. NCBI Datasets provides a novel data delivery mechanism that delivers data packages that combine sequence and metadata stored in multiple NCBI databases for a single request. Providing data as part of a data package offers several advantages over data delivery as standalone files. First, bundling metadata with data promotes the FAIR principles of interoperability, accessibility, and reuse. Every downloaded data package includes sufficient descriptive metadata to understand the contents, enabling confident reuse in any downstream analysis and publication citation. Second, by delivering the data in zip archives, related file types such as protein and corresponding transcript sequences are bundled together, making it more straightforward for users to find consistency in the data. Zip archives also allow compressed file sizes, making downloads faster and more reliable. Lastly, by delivering metadata alongside the data, data packages ensure that users can access information for citation purposes. This practice supports proper attribution and enhances the reproducibility and transparency of research findings.

**Fig. 2:**
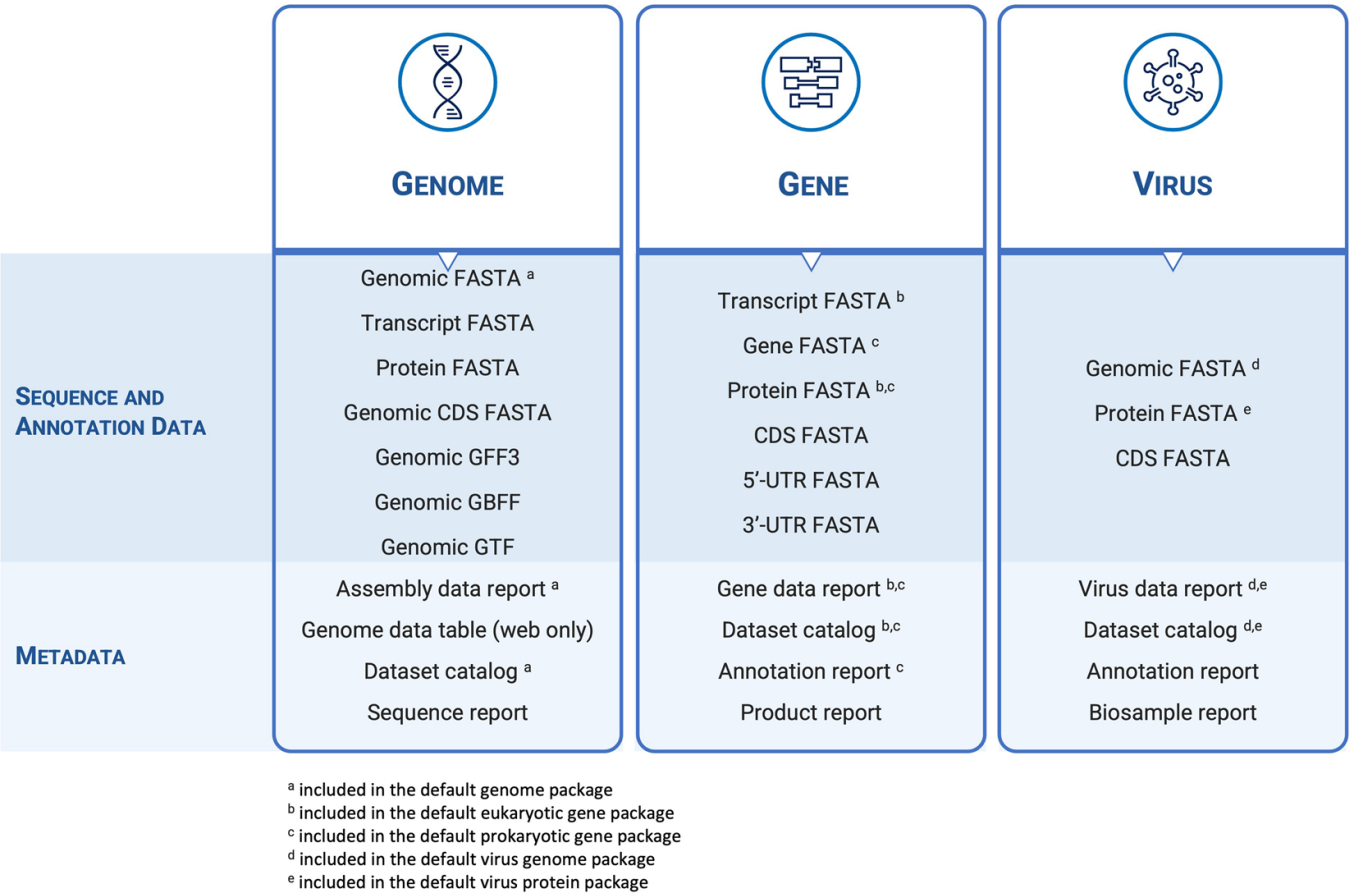
Types of data packages: Three main categories of data packages are currently available: genome, gene, and virus. Users can customize the contents of any data package. Detailed information about each data package, including a list of available files and descriptions of each file, is available in NCBI Datasets documentation under Data packages (https://www.ncbi.nlm.nih.gov/datasets/docs/v2/reference-docs/data-packages/).

Currently, NCBI Datasets offers three types of data packages: gene, genome, and virus data packages (Fig. 2). The contents of these data packages vary depending on the data package type, details of the user request, and file availability. For example, genome data packages will only include annotation files (e.g., GFF3 or GTF) if the genome has been annotated and the user has requested that file at the time of download. All data packages include one or more data reports, containing metadata, as well as a dataset catalog that describes the files included in the package.

### Genome package

Genome data packages are available for the nearly two million eukaryotic and prokaryotic assembled genomes and encompass genomic, transcript, and protein sequences, as well as genome annotation files for all GenBank and NCBI RefSeq genomes stored in the NCBI Assembly database^6^. Annotation files are available in GFF3, GTF, or GBFF formats. Annotation files and sequence files for transcripts, coding sequences (CDS) and proteins are only available for genomes annotated by NCBI RefSeq or GenBank submitters. The package also includes two distinct data reports, an assembly data report, and a sequence report. The assembly data report (assembly_data_report.jsonl) combines metadata and other information from NCBI’s Assembly, Taxonomy, BioSample, Gene, and BioProject databases, as well as the NCBI RefSeq annotation details. The sequence report (sequence_report.jsonl) contains detailed metadata for the individual contig, scaffold, or chromosome sequences that comprise the assembled genome. While including the sequence report in the package is optional, the assembly data report is *always* included.

### Gene package

Gene data packages center around genes and incorporate gene, transcript, and protein sequences. These packages are exclusive to genes present in the RefSeq database, which encompasses genes annotated by the NCBI RefSeq eukaryotic and prokaryotic annotation pipelines, as well as user-submitted annotations that have been incorporated into the RefSeq database^7^.

For eukaryotic genes, selected areas of transcribed sequences – including the 5′ and 3′ untranslated regions (UTRs) and coding sequences (CDS) – can also be accessed as individual files. RefSeq annotated eukaryotic, and a subset of RefSeq prokaryotic genes from the reference assemblies, are described by two data reports. The gene data report (data_report.jsonl) contains curated metadata found in NCBI Gene (https://www.ncbi.nlm.nih.gov/gene). The gene product report (product_report.jsonl) details the gene products (transcripts and proteins), including annotation information such as exon coordinates on RefSeq genomes.

Similarly, all prokaryotic RefSeq genes have two data reports: a gene data report (data_report.jsonl) comprising metadata from the NCBI RefSeq non-redundant protein accession (WP_) and an annotation report (annotation_report.jsonl) detailing all the genomes where the non-redundant RefSeq protein has been annotated.

Gene packages for a set of orthologs are also available. The NCBI’s Eukaryotic Genome Annotation pipeline (EGAP) calculates the ortholog groups. This calculation relies on protein sequence similarity and local synteny information^8^. Orthologs, homologous genes descended from a common ancestor, are determined by comparing an annotated genome with a reference genome. The sets of pairwise orthologs are then tracked as a group. The choice of reference genome varies: *Homo sapiens* (human) is used for most vertebrates other than fish; *Danio rerio* (zebrafish) is used for fish; and *Drosophila melanogaster* (fruit fly) is used for insects. It is important to note that only the genes present in the NCBI Gene database are included in these ortholog calculations.

### Virus package

NCBI Datasets created a separate virus data package to meet the specific data requirements of the public health and virus research communities. Virus data packages include data for viral genomes available in the NCBI Virus database, an integrative, value-added resource designed to support the retrieval, display, and analysis of a curated collection of virus sequences and large sequence datasets (https://www.ncbi.nlm.nih.gov/labs/virus/vssi/). These packages include genomic, coding sequence (CDS), and protein sequences. Like other packages, a primary metadata report is included. Users can choose to include additional reports detailing the annotation of genes and proteins for each viral genome and a comprehensive BioSample report. This data can be conveniently retrieved by accession number or taxonomic name.

## Discussion

NCBI Datasets has only been around for a few years, but it has significantly impacted how data is discovered and downloaded from NCBI. During the COVID-19 pandemic, the number of SARS-CoV-2 assembled genomes submitted to NCBI increased from a few thousand to over eight million, presenting an unprecedented challenge to deliver a set of genomes and metadata. NCBI Datasets, working closely with the CDC, other public health groups, and the NCBI Virus team, provided a data delivery method that gave the global community access to daily updates to the massive collection of SARS-CoV-2 assembled genomes^9^. NCBI Datasets provides easy download options for the NCBI Pathogen’s MicroBIGG-E and Reference Gene Catalog interfaces and scalable access to the approximately 1.8 million prokaryotic genomes^10^. Finally, NCBI Datasets has made it significantly easier for the eukaryotic research community to find data, connect to tools such as NCBI’s Genome Data Viewer (GDV) and BLAST, and download sequence and metadata supporting NIH’s Comparative Genomics Resource (CGR), a multi-year National Library of Medicine (NLM) project to maximize the impact of eukaryotic research organisms and their genomic data resources to biomedical research^11^.

### Future plans

NCBI Datasets is a resource that remains under active development that will expand and strive to include a wider range of data types and further improve data accessibility. We will continue exploring innovative data delivery methods to better accommodate the growing volume of data submitted to NCBI. We understand that this will require many in the biological research community to learn new ways of accessing data, and we are committed to making this access as user-friendly as possible and supporting the growing community of data scientists. We want to ensure that the services we develop help the biological research community make the most of the vast amounts of data stored in public repositories. Active engagement with the biological research community is a crucial component of the effort. By collaborating directly with the researchers and users of the data, the NCBI Datasets team can gain a better understanding of their needs, enabling NCBI Datasets to provide services that are directly aligned with the requirements of the community.

## Methods

### Metadata formats and schemas

NCBI Datasets delivers metadata as data report files in JSON and JSON Lines formats (https://jsonlines.org). JSON and JSON Lines formats are simple and easy-to-use formats with strong tooling support. Additionally, these formats balance human readability and convenience with machine readability and operability. A companion tool called *dataformat* (see *Command-line tools* section below) is also available to facilitate access to the information in the data reports by converting them to comma-separated values (.csv) or Excel^®^ (.xlsx) formats. Compared to tabular formats, JSON and JSON Lines formats maintain the hierarchical organization of data, preserve relationships between fields, and are extensible, in that new fields or sets of fields can be easily added without introducing breaking changes. Although all metadata is provided in either JSON or JSON Lines formats by default, it is recognized that many users prefer working with data in a tabular format for browsing and analysis purposes. To address this, we provide tools to convert JSON Lines reports to tabular formats and offer table downloads directly from our web interfaces. For a comprehensive understanding of each data report, including field descriptions and example values, detailed schemas are available on our documentation pages under Data report schemas (https://www.ncbi.nlm.nih.gov/datasets/docs/v2/reference-docs/data-reports/). All fields in the data reports are included in the schema pages.

### How does NCBI Datasets support data exploration and downloads?

To provide maximum flexibility in data access for biologists and bioinformaticians, NCBI Datasets offers a variety of interfaces to browse and download data, including web pages, command-line tools, and OpenAPI (https://www.openapis.org/). Most users will find that web pages and command-line tools, or a combination of both, will best serve most data exploration and download goals. The NCBI Datasets API also serves as the primary data source for both the web pages and command-line tools, ensuring data consistency regardless of the interface used. Together, these support the FAIR principles of findability and accessibility through easy discoverability, scalable download options, extensive metadata, and interoperable formats.

### Web interface

**Fig. 3:**
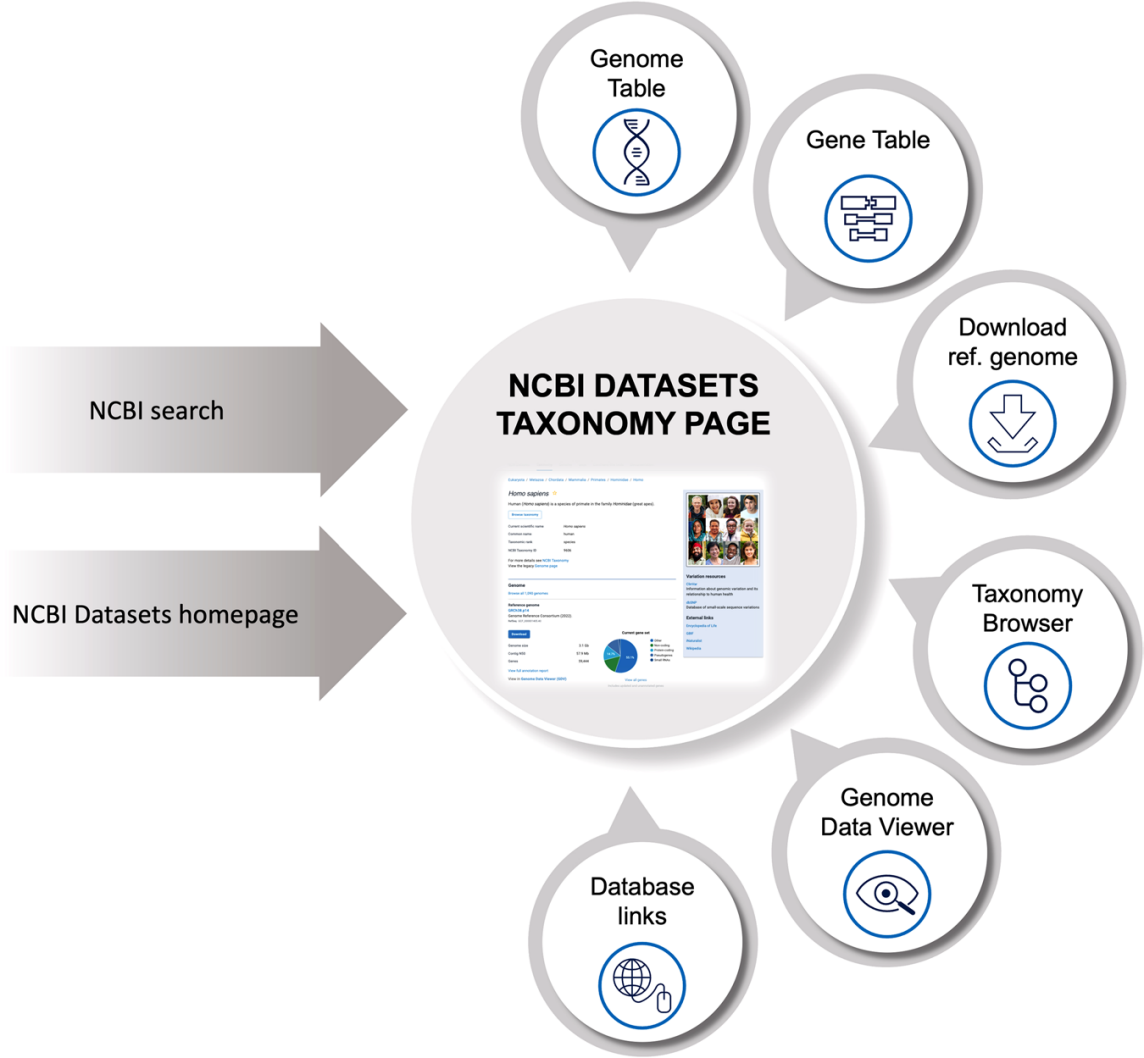
Organism-focused data access: The NCBI Datasets taxonomy web page provides access to NCBI sequence and metadata data for that organism, including tabular views or assembled genome and annotated genes, download options and links to data in other NCBI databases.

The NCBI Datasets web interface (https://www.ncbi.nlm.nih.gov/datasets) offers a user-friendly organism-centric experience for searching, browsing, and downloading data from across the NCBI sequence databases (Fig. 3). Users can enter a species name from the NCBI Datasets homepage to navigate to a taxonomy page representing that species. The taxonomy pages act as a gateway to NCBI data available for each node of the taxonomic tree, connecting to gene and genome pages relevant to that taxonomic node, as well as to related taxa.

NCBI Datasets taxonomy pages replace the legacy Entrez Genome pages, providing basic taxonomic information and a high-level look at the gene and genome data available for that taxon. The taxonomy pages conveniently link to the NCBI Datasets taxonomy browser, genome table, genome pages, and gene table for annotated genomes. Additionally, they provide links to data stored in other NCBI databases not yet incorporated into NCBI Datasets.

NCBI Datasets gene and genome tables allow users to simultaneously browse large numbers of records, with links to individual records and options to download data. The gene table allows browsing of genes for a species, with each row representing a single gene, and columns representing various metadata fields. The “Actions” column links the Genome Data Viewer (https://www.ncbi.nlm.nih.gov/gdv/) and NCBI ortholog pages (https://www.ncbi.nlm.nih.gov/gene/59272/ortholog/?scope=7742). The genome table allows users to browse assembled genomes, with each row representing a genome, and columns representing key metadata. It offers options to download data packages and tables. The “Actions” column in the table provides links to the NCBI Datasets genome pages, BLAST, and the Genome Data Viewer (GDV). NCBI Datasets genome pages represent individual assembled genomes, replacing the legacy Entrez Assembly pages. Similar to the legacy pages, the NCBI Datasets genome pages describe single genomes and provide options for downloading data. In contrast to the legacy pages, the new genome pages consolidate information from multiple NCBI databases (such as Assembly, BioProject, and BioSample) on a single page. In addition, command-line and curl commands are provided at the top of each page, enabling users who browse on the web to easily get data in a terminal environment. This also offers an accessible introduction for those unfamiliar with the command-line.

Finally, the taxonomy browser allows the easy exploration of organisms within their taxonomic context, visualizing available assembled genomes for different ranks. As NCBI Datasets grows, we will continue to add a taxonomic view to additional data types. All data visible on NCBI Datasets web pages can be downloaded via the blue download buttons.

### Command-line tools

The command-line tools offer programmatic access to all data available on the NCBI Datasets web pages. With the increasing complexity of metadata and the growing volume of sequence data, robust programmatic access is crucial for biologists to fully utilize NCBI data.

**Fig. 4:**
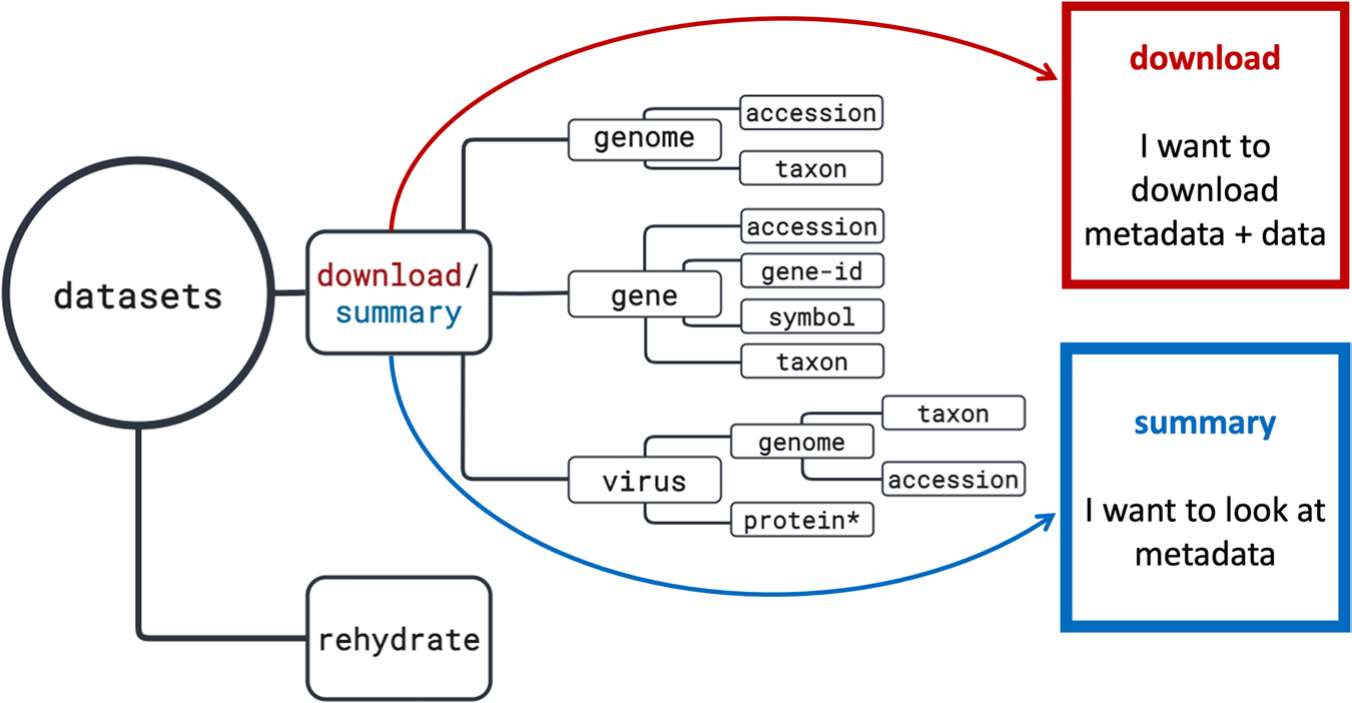
Organization of the *datasets* command-line tool: The *datasets* command-line tool can be used to browse and download NCBI Datasets data packages. There are two main subcommands: “download” for retrieving data packages and “summary” for displaying metadata. Each subcommand has multiple flags to help narrow data packages to the desired genomes or genes of interest. For an overview of *datasets*, *dataformat*, and installation instructions, see our Command-line tools documentation (https://www.ncbi.nlm.nih.gov/datasets/docs/v2/download-and-install/). (*) virus protein restricted to download of SARS-CoV-2 proteins.

We offer two command-line tools: *datasets* and *dataformat*. The *datasets* tool allows users to download genome, gene, and virus data packages. The companion tool, *dataformat*, converts content from the metadata reports that use the JSON Lines format into more user-friendly tabular formats (Fig. 4). The command-line tools are available on Mac, Linux, and Windows platforms via NCBI or may be installed using conda (https://anaconda.org/conda-forge/ncbi-datasets-cli). Our tools are accessible even to users new to the command-line environment as they are intuitive, well-organized, and flexible.

NCBI Datasets command-line tool syntax is characterized by commands and nested subcommands, followed by query terms such as taxonomic names, genome, gene or BioProject accessions. These subcommands are named using plain language terms that are easily recognizable such as “gene” or “genome.” This is a distinct departure from NCBI E-utilities^12^, which requires knowledge of NCBI’s many databases.

**Fig. 5:**
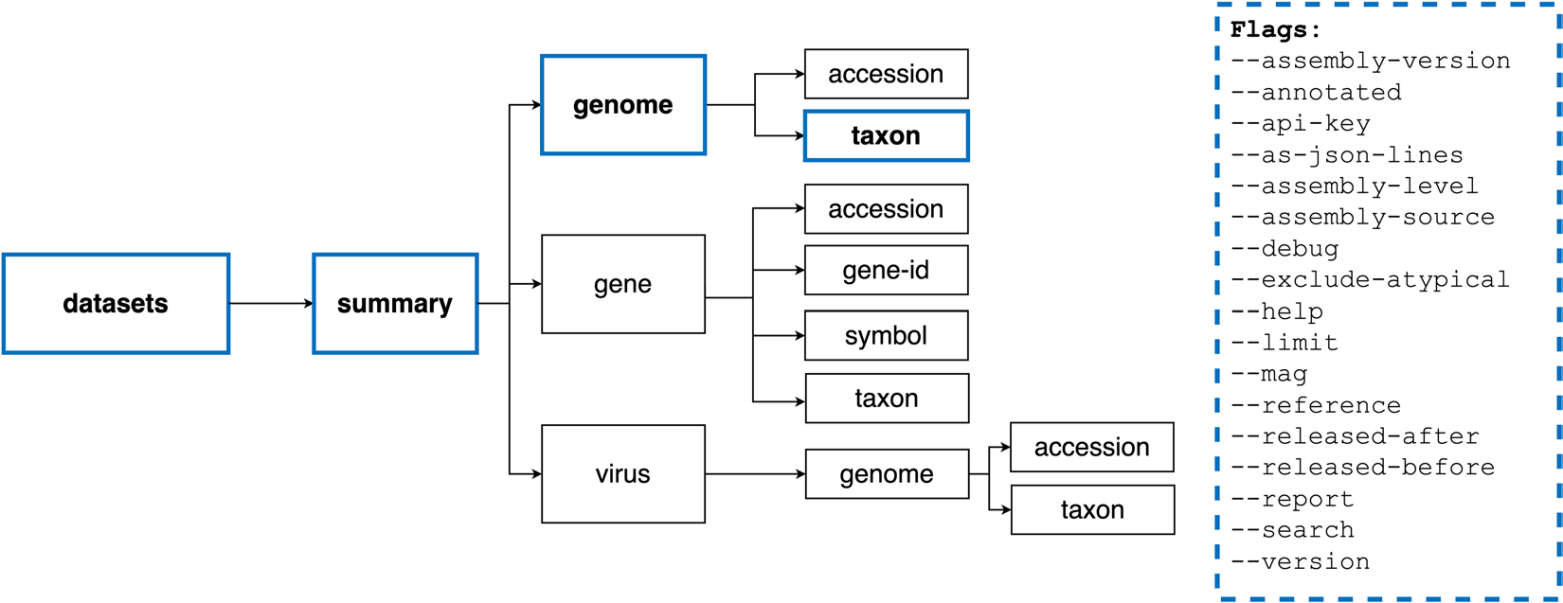
The *datasets* summary command: Diagram of the *datasets* command-line tool syntax for getting metadata for one or more genomes by taxon name.

The *datasets* command-line tool features two major top-level commands: “download” and “summary” (Fig. 4). The download command returns the NCBI Datasets data package as a zip archive, while the summary command prints metadata to the terminal screen. Subcommands are used to specify the type of data requested. Using subcommands, the datasets tool provides context-specific help, including a brief overview of subcommands and descriptions of the corresponding available flags. For example, to get more information about how to obtain genome metadata for a particular taxon of interest, the command datasets summary genome taxon–help returns a list of available flags, including options to restrict to only annotated or reference genomes, or genomes released during a specified date range (Fig. 5).

Query terms and identifiers that can be used to find data in the web interface can also be used with the command-line tool. For example, genome data can be queried using common or scientific taxonomic names, such as “human” or “*Mus musculus*,” or using assembly (e.g., GCF_000001405.40) or BioProject (e.g., PRJNA705675) accessions. Similarly, gene data can be queried by common and scientific species names, gene symbols or aliases, and transcript or protein accessions.

**Fig. 6:**
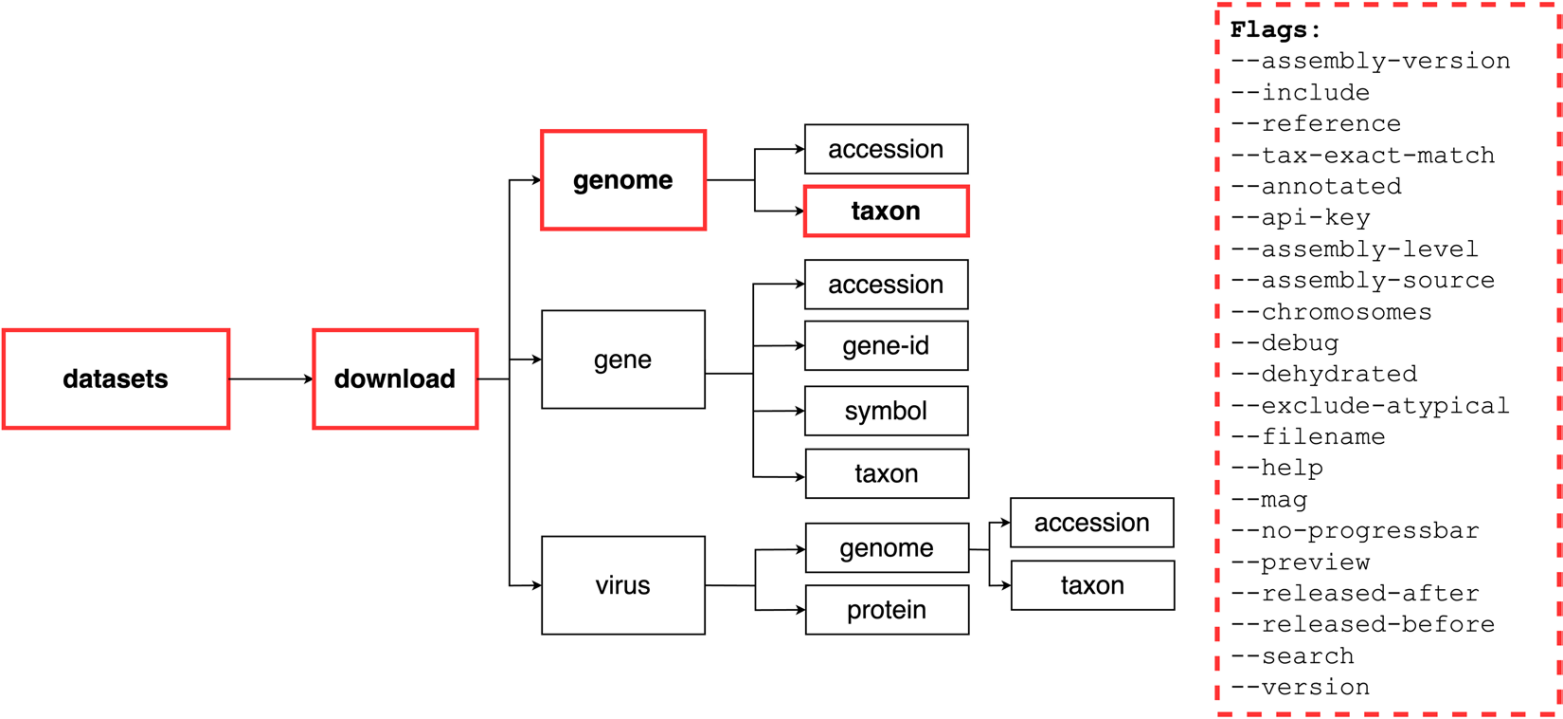
The *datasets* download command: Diagram of the *datasets* command-line tool syntax for downloading a genome data package for one or more genomes by taxon name.

For example, the following command prints genome metadata to the screen describing the human reference genome, GRCh38: datasets summary genome taxon human --reference. In contrast, the command datasets download genome taxon siluriformes --annotated --include protein results in the download of a genome data package containing protein sequences for annotated genomes from the order Siluriformes or catfish (Fig. 6).

**Fig. 7:**
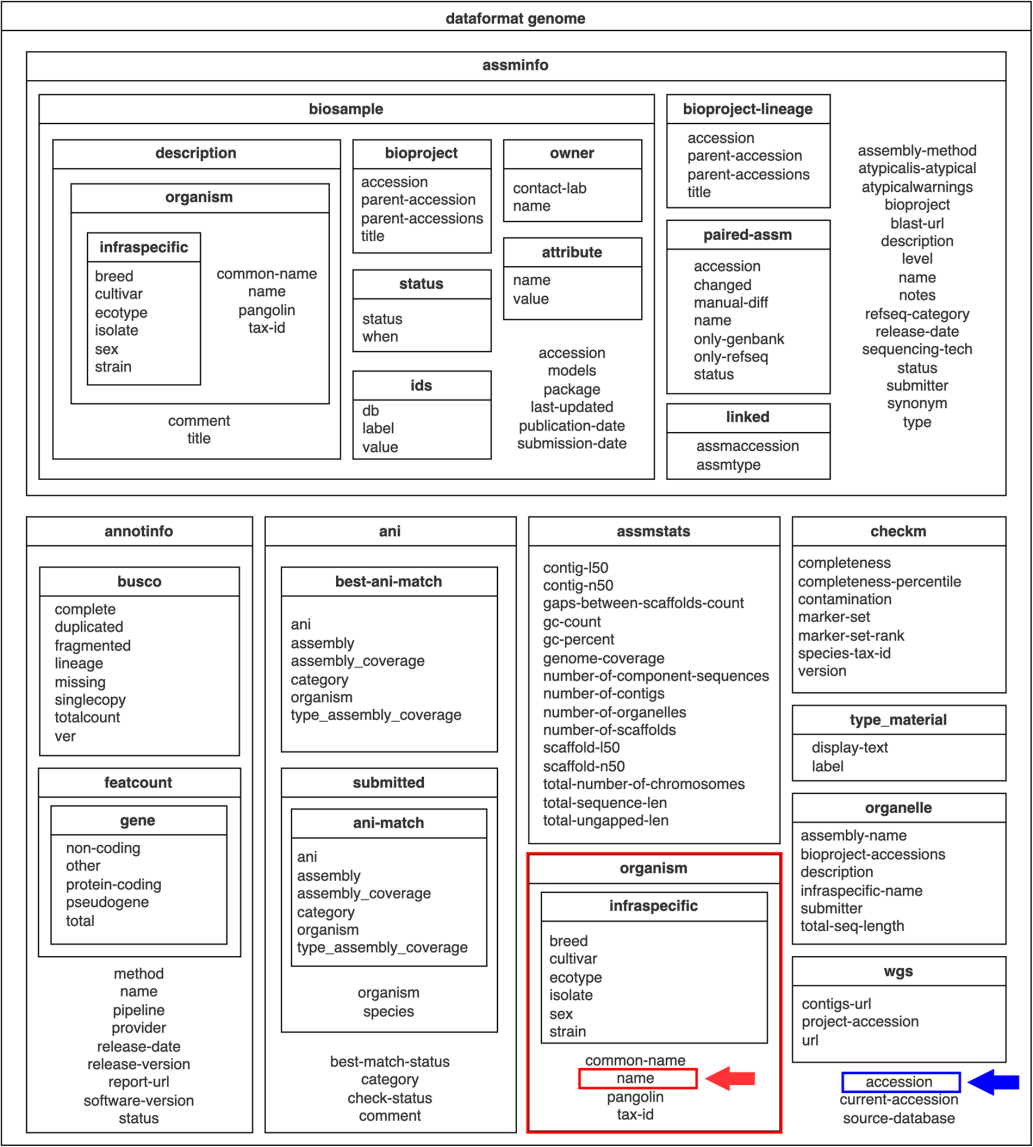
Organization of the assembly_data_report.jsonl data report. Select metadata can be retrieved from the data report using the *dataformat* command line tool. In this example, the organism name (red) and assembly accession (blue) can be extracted from the data report to generate a two-column tabular file.


The *dataformat* command-line tool converts JSON Lines metadata to either a tabular (.tsv) text format or an Excel spreadsheet (.xlsx) with a set of user-specified fields. The layout of the JSON Lines metadata report is illustrated in Fig. 7. Each ‘box’, or line, in the JSON Lines format, encapsulates metadata pertinent to a genomic sequence, with the capability to nest further detailed ‘boxes’ of metadata within each line. Metadata can be piped directly from *datasets* to *dataformat* or, alternatively, the path to the relevant metadata can be passed as input. For instance, a simple two-column table describing mouse genomes released during a three-month period in 2023 can be created with the following command:

datasets summary genome taxon 'mus musculus'--released-after 4/1/2023 --released-before 7/1/2023--as-json|-lines |dataformat tsv genome --fields organism-name,accession


This returns the following output:


Organism Name Assembly AccessionMus musculus GCA_030265425.1Mus musculus GCA_949316305.1Mus musculus GCA_949316315.1


A list of available fields can be accessed through the CLI help menu or web documentation. For example, to find a list of fields available for generating tables describing genome data, refer to the *dataformat* (https://www.ncbi.nlm.nih.gov/datasets/docs/v2/reference-docs/command-line/dataformat/tsv/dataformat_tsv_genome/) reference page.

## Data Availability

**NCBI Datasets documentation.** Effective documentation plays a crucial role in facilitating the utilization of bioinformatic tools, particularly for users new to programmatic access. The documentation pages serve as an ideal starting point, offering clear instructions and options for downloading and installing the command-line tools (https://www.ncbi.nlm.nih.gov/datasets/docs/v2/download-and-install/). How-to guides (https://www.ncbi.nlm.nih.gov/datasets/docs/v2/how-tos/) enable users to quickly grasp command structures, flag usage, and search filtering techniques. For complex tasks, longer training resources and tutorials (https://www.ncbi.nlm.nih.gov/datasets/docs/v2/tutorials/) provide step-by-step instructions. The project remains under active development, with release notes on GitHub (https://github.com/ncbi/datasets/releases) to keep users updated on the latest enhancements to NCBI Datasets services. GitHub also provides a platform for users to give feedback on bugs and propose enhancements. **Using NCBI Datasets in Galaxy.** The Galaxy project’s main server, an open-source, web-based platform for data-intensive biomedical research, now features integration of NCBI Datasets’ genome and gene services (The Galaxy Community, 2022)^13^. This enhancement enables users to directly access up-to-date sequences and metadata for all assembled genomes and RefSeq genes from the NCBI databases within the Galaxy environment. Furthermore, it facilitates the direct export of files from the NCBI Datasets web pages to Galaxy. By developing NCBI Datasets in such a way that it can be integrated into community platforms such as Galaxy, we are helping democratize access to extensive publicly available biological sequence data and metadata, offering significant benefits, especially to users without the capacity for large-scale computational tasks due to limited server space.

## Code availability

Source code for the Datasets command line tooling is in the process of being made available on GitHub at https://github.com/ncbi/datasets. Instructions on how to use the tooling is currently available at that same location. Source code for the Datasets front end interface and backend servers are not provided publicly.
